# Mild Hydrothermal Synthesis of 11Å-TA from Alumina Extracted Coal Fly Ash and Its Application in Water Adsorption of Heavy Metal Ions (Cu(II) and Pb(II))

**DOI:** 10.3390/ijerph19020616

**Published:** 2022-01-06

**Authors:** Jingjie Yang, Hongjuan Sun, Tongjiang Peng, Li Zeng, Xin Zhou

**Affiliations:** 1 Key Laboratory of Ministry of Education for Solid Waste Treatment and Resource Recycle, Southwest University of Science and Technology, Mianyang 621010, China; yangjingjie007@mails.swust.edu.cn (J.Y.); pengtongjiang@swust.edu.cn (T.P.); zengli6041@mails.swust.edu.cn (L.Z.); 18355412280@sina.cn (X.Z.); 2 Institute of Mineral Materials & Application, Southwest University of Science and Technology, Mianyang 621010, China; 3 Mian Yang City College, Southwest University of Science and Technology, Mianyang 621010, China

**Keywords:** residue after extracting aluminum from CFA (REA), synthetic tobermorite, heavy metals, copper, lead, adsorption, kinetics study

## Abstract

Non-biodegradable copper (Cu) and lead (Pb) contaminants in water are highly toxic and have series adverse effects. Therefore, it is very important to extract heavy metals from wastewater before it is discharged into the environment. Adsorption is a cost-effective alternative method for wastewater treatment. Choosing a low-cost adsorbent can help reduce the cost of adsorption. In this study, conversion of reside after extracting aluminum (REA) produced by sub-molten salt method transform high-alumina coal fly ash (CFA) into 11Å-tobermorite to adsorb Cu(II) and Pb(II) from aqueous solutions at room temperature. The synthesis of the adsorbent was confirmed using scanning electron microscope (SEM), X-ray diffractometer (XRD) and Brunauer–Emmett–Teller (BET) method surface analysis. To study the adsorption characteristics, factors such as initial Cu(II) and Pb(II) concentration, pH, contact time, adsorption characteristics and temperature were investigated in batch mode. The maximum adsorption capacity of Cu(II) and Pb(II) was 177.1 mg·g^−1^ and 176.2 mg·g^−1^, respectively. The Langmuir adsorption model was employed to better describe the isothermal adsorption behavior and confirm the monolayer adsorption phenomenon. The pseudo-second-order kinetic model was used to highlight Cu(II) and Pb(II) adsorption kinetics. Thermodynamic analysis indicated the removal Cu(II) and Pb(II) by TA-adsorbent was a nonspontaneous and exothermic reaction. The obtained results are of great significance to the conversion of industrial waste to low-cost adsorbent for Cu(II) and Pb(II) removal from water.

## 1. Introduction

Water pollution is a major problem humankind faces today [1], large-scale wastewater is inevitable generated by industrial and economic development and places a lot of strain on ecosystem [2,3]. Heavy metals have been recognized as highly toxic contaminants owing to their highly pathogenic and bioaccumulation throughout the food chain [4]. Among the heavy metals, copper (Cu(II)) and lead (Pb(II)) are of particular interest as they are extensively produced in human activities and have been released into the aquatic environment [5,6]. According to the values announced by the World Health organization (WTO) and Environmental Protection Agency (EPA), the maximum permissible limits of Cu(II) and Pb(II) in drinking water are 1 mg·L^−1^ [7] and 0.05 mg·L^−1^ [8], respectively. Once these metals are ingested beyond the maximum permissible limits, they may result in mutagenic and carcinogenic effects could cause further damage to multiple systems and organs of the human body, or even death [9]. Nowadays, remediate Cu(II) and Pb(II) contaminated water has been developed by various techniques, such as chemical precipitation, flocculation, ion exchange, membrane technology and permeable reactive barriers (PRBs) [10], among them, adsorption has low initial capital and maintenance costs and applicable to many technologies [11].

Over the last decade, many studies have focused on utilization of unique Coal fly ash (CFA) distribution in northern Shanxi and western part of Inner Mongolia China as the potential substituted resource of bauxite for the alumina industry, because it contains 40-50% alumina [12,13,14,15]. Among these efforts, the hydro-chemical process has emerged as a more promising alternative [16]. the process was first proposed by researchers in the former Soviet Union, which is believed to be an effective method for “Al-rich” waste product, such as red mud (RM) [17], high-alumina coal fly ash (HAFA) [12] and low-grade bauxite. During the process, the silicon-bearing components react with the slaked lime to generate a new crystal phase (NaCaHSiO_4_) and enter the solid phase, where alumina is extracted from HAFA under high temperature and pressure. Sun proposed a mild hydro-chemical process for the extraction of alumina from HAFA [18]. The alumina extraction efficiency reached above 90% under optimal conditions. However, it is known that NaCaHSiO_4_ exhibits strong alkalinity and can potentially damage agricultural land and groundwater if not appropriately treatment [18]. Previous studies have indicated that NaCaHSiO_4_ is easily digested and converted to tobermorite adsorbent (TA) in diluted alkaline solution [19,20]. Additionally, the physiochemical properties of TA make it a potential adsorbent.

Tobermorite is a layer-lattice calcium silicate hydrate mineral, which consists of infinite layers of Ca-O polyhedra, with wollastonite-type silicate chains condensed on both sides of Ca-O polyhedra [21]. Three different individual tobermorite (14 Å, 11 Å and 9 Å tobermorite) can be defined owing to their c-axis length [22], among these, 11Å-tobermorite is structurally stable at ambient temperature and the most important mineral of the tobermorite family. Apart from their widespread application in the building materials and refractory materials, tobermorite is nowadays extensively applied in organic or inorganic effluent treatment due to its high specific surface area and ion-exchange capacity [23]. In previous studies, 11Å-tobermorite has been synthesized from pure chemical sources. If industrial wastes serve as the sources of calcium or silicon, the synthesis cost of TA can be further reduced. Herein, alumina-extracted residue (AER) from the mild hydro-chemical process, whose principal crystalline constituents were NaCaHSiO4, was used to synthesize 11Å-TA via hydrothermal method. Different Ca/Si molar ratio, hydrothermal temperature and time were designed as the modification parameters. Furthermore, prepared 11Å-TA was applied to Cu(II) and Pb(II) removal from water solutions, where the removal mechanism and kinetics of Cu(II) and Pb(II) were studied in detail. This work not only reduces the cost of Cu(II) and Pb(II) removal but also ameliorates issues related to solid waste disposal.

## 2. Materials and Methods

### 2.1. Raw Materials

**Figure 1:**
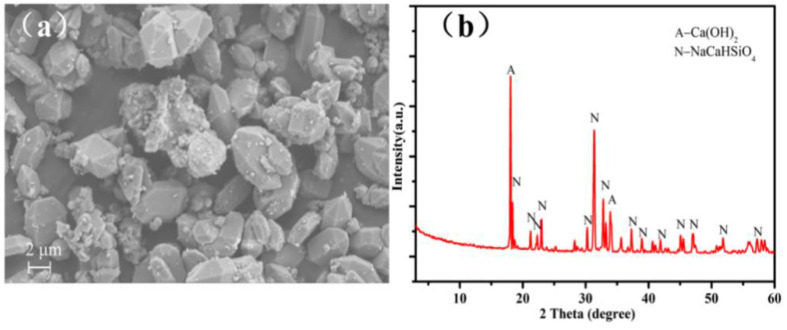
Scanning electron microscope (SEM )image (**a**) and X-ray diffractometer (XRD) pattern (**b**) of alumina-extracted residue (AER).

AER obtained using hydrothermal process from high-alumina coal fly ash (HCFA) (collected from the Inner Mongolia Da Tang Thermal power plant, Hohhot, China ). The operation conditions of AER synthesis were previously reported [24]. X-ray fluorescence (XRF, PANalytical, Eindhoven, Netherlands) was used to determine the chemical composition of AER, (Table 1), where SiO_2_ content was 35.57% and Na_2_O content was 17.91%. Therefore, the high alkalinity of AER without treatment generates environmental pollution. In contrast, exhibits AER potential recycling value for the recovery of alkali, and is a source of silicon and calcium. AER also contains trace quantities of various metal oxides such as Fe_2_O_3_, Al_2_O_3_, TiO_2_ and MgO. Scanning electron microscope (SEM) analysis of AER is shown in Figure 1a. X-ray diffractometer (XRD) pattern of AER (Figure 1b) shows that the main phases correspond to NaCaHSiO_4_ and Ca(OH)_2_. The remaining chemical reagents (analytical grade) were supplied by KULONG Chemical Reagent Co. Ltd. (Chengdu, China). Deionized water was used in all experiments.

### 2.2. Preparation of Adsorbents

11Å-TA from AER was synthesized by hydrothermal synthesis, where AER was sufficiently alkaline to effect the decomposition of itself without addition of a strongly basic reaction medium. AER molar ratio Ca/Si was approx 1.1 (Table 1). However, the optimal molar ratio of Ca/Si was 0.83, which was calculated from the ideal composition of 11Å-TA (5CaO·6SiO_2_·5H_2_O). Hence, the molar ratio of Ca/Si was adjusted by mix ratio of fumed silica and AER, which also allowed to study the effect of different mix mass ratio of fumed silica and AER on the hydrothermal product, with the designed molar ratio of Ca/Si was being 0.8–1.1. In all batches, the liquid-solid ratio was 25 mL·g^−1^. The slurry was transferred to a 100 mL polytetrafluoroethylene (PTFE)-lined Ni steel autoclave after mixing, the autoclave was sealed and finally placed in an oven (WGZ-9040, ever briGht medical treatment instrument Co. Ltd., Beijing, China) to conduct hydrothermal experiments, which were performed over specific times at different temperatures. The overall reaction conditions are summarized in Table 2. When the hydrothermal process was finished, the products were filtered after cooling to room temperature and washing to neutral pH. The synthesized samples were collected and dried at 103 °C to constant weight for characterization.

### 2.3. Adsorption Batch Tests

In order to explore the adsorption and potential of synthetic 11Å-(TA) to remove Cu(II) or Pb(II), gradient solutions of metal ions between 200 mg·L^−1^ and 2000 mg·L^−1^ were prepared by subsequent dilution of the stock solutions using deionized water. The stock solution (4000 mg·L^−1^) of Cu(II) and Pb(II) ions were prepared by dissolving CuSO_4_·5H_2_O and Pb(NO_3_)_2_ in deionized water, respectively. According to the solubility product principle, in view of the difference between K_sp_[Pb(OH)_2_] (25 °C) and K_sp_[Cu(OH)_2_] (25 °C), Cu(II) and Pb(II) began to precipitate at approx pH 5.3 and 6.6, respectively [25]. Therefore, the initial pH value of Cu(II) and Pb(II) solution were adjusted to 5.0 in the subsequent experiment using dilute HNO_3_ (0.1 M) and NaOH (0.1 M) solutions. 

A batch equilibrium method was applied to the removal experiments. Typically, 0.1 g of TA was added into a 100 mL centrifuge tube. Then, 25 mL of simulated contaminated water (Cu(II) or Pb(II)) was added and the tube was subjected to a thermostatic oscillator (SHA-B, LiChen Co. Ltd., Shanghai, China) at 200 rpm for 12 h at room temperature (25 ± 2 °C). After adsorption, the slurry was centrifuged for 180 s at 6000 rpm. Liquid supernatant was withdrawn using an injection syringe (25 mL, Kelun Co. Ltd., Chengdu, China) and then filtered through a 0.45 μm polyethersulfone membrane filter (JinTeng Co. Ltd., Tianjin, China). Then filtrate was immediately diluted 10 times with distilled water. The amount of metal cations was determined using an inductively coupled plasma mass spectrometer (ICP, ICAP6500, Thermo Fisher Scientific, Waltham, MA, USA). To determine the amount of Cu(II) and Pb(II) absorbed on TA, the following equations were used:

(1) $$q_{e}=\frac{(C_{0}{-C}_{e})\times V}{M}$$

(2) $$\%\mathrm{Removal}=\frac{\left(C_{0}-C_{e}\right)}{C_{0}}\times 100$$

where q_e_ is the adsorption capacity of absorbent for heavy metal ions (mg·L^−1^); C_0_ is the initial concentration of the heavy metal ions (mg·L^−1^) and C_e_ is the equilibrium concentration of heavy metal ions (mg·L^−1^); V and m are the volume of solution (L) and the mass of the dry adsorbent (g), respectively. All glass and plastic containers in the experiment were soaked in 0.5 M nitric acid solution for 24 h and then washed repeatedly with distilled water and dried under 70 °C.

In order to observe the effect of contact time on the adsorption of heavy metals on the obtained TA, all experiments were performed with 0.1g of TA suspended in 25 mL of Cu(II) (1000 mg·L^−1^) or Pb(II) (1000 mg·L^−1^) solutions at initial pH 5.0 ± 0.1. The effect of contact time on the adsorption rate were carried out by performing the adsorption experiments at pre-determined time intervals (5, 20, 40, 60, 90, 120, 180, 240, 300 and 360 min). The samples were collected at various intervals of time in order to monitor the reaction in kinetics.

To test the effects of solution pH on the adsorption of heavy metals on TA, 0.1 g TA was added to 25 mL of 200 mg·L^−1^ Cu(II) or Pb(II) solution at pH 1.0–6.0. pH values of the solutions were measured using a pH meter (PHS-3C, INESA scientific instrument Co. Ltd., Shanghai, China). The residual heavy metal concentration in the upper clear liquid was detected by ICP. 

### 2.4. Characterization Techniques

The crystal structures were determined using X-ray diffractometer (XRD, Ultima IV, Rigaku Co. Ltd., Tokyo, Japan) with a Cu K_α_ radiation (λ = 0.154 187 nm) operation at 40 kV and 40 mA. 2*θ* scans were performed from 3° to 80° at a rate of 15°·min^−1^. The chemical compositions were analyzed using X-ray fluorescence analyzer spectrometer (XRF, PANalytical, Eindhoven, The Netherlands). The specific surface area of the obtained material was measured using surface area analyzer (Autosorb-iQ, Quantachrome, Boynton Beach, FL, USA) and evaluated by measuring the isothermal adsorption of N_2_ based on the single-point Brunauer–Emmett–Teller (BET) method. The microstructural development and chemical characteristics of samples were studied using a scanning electron microscope (SEM, Zeiss Instruments, Oberkochen, Germany) equipped with energy dispersive spectroscopy (EDS, Oxford, UK). The zeta potentials of the obtained TA adsorbent were conducted by Nano zeta sizer (Zs90, Malvern Instruments, Malvern, UK) in electrophoretic light scattering mode. The concentration of the metal ions in solution was measured via an inductively coupled plasma mass spectrometer (ICP, Thermo Fisher Scientific Co. Ltd., Waltham, MA, USA).

## 3. Results and Discussion

### 3.1. Characterization of TA from AER

**Figure 2:**
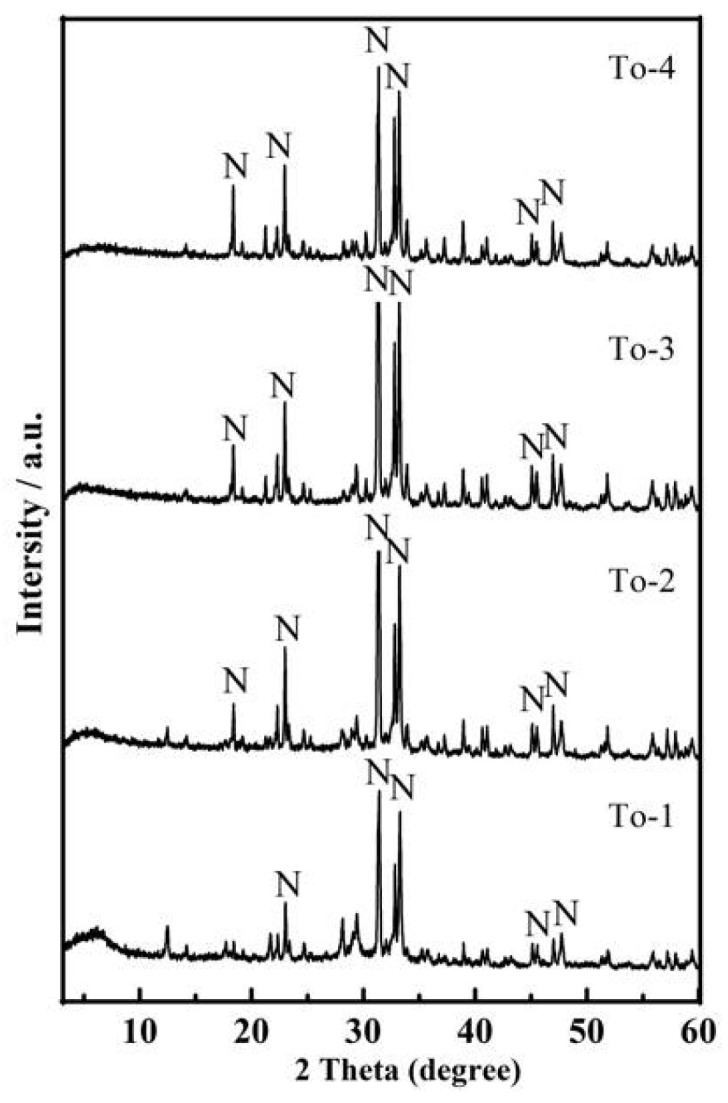
XRD pattern of products synthesized at different Ca/Si molar ratio at 160 °C for 12 h. N—NaCaHSiO_4_.

**Figure 3:**
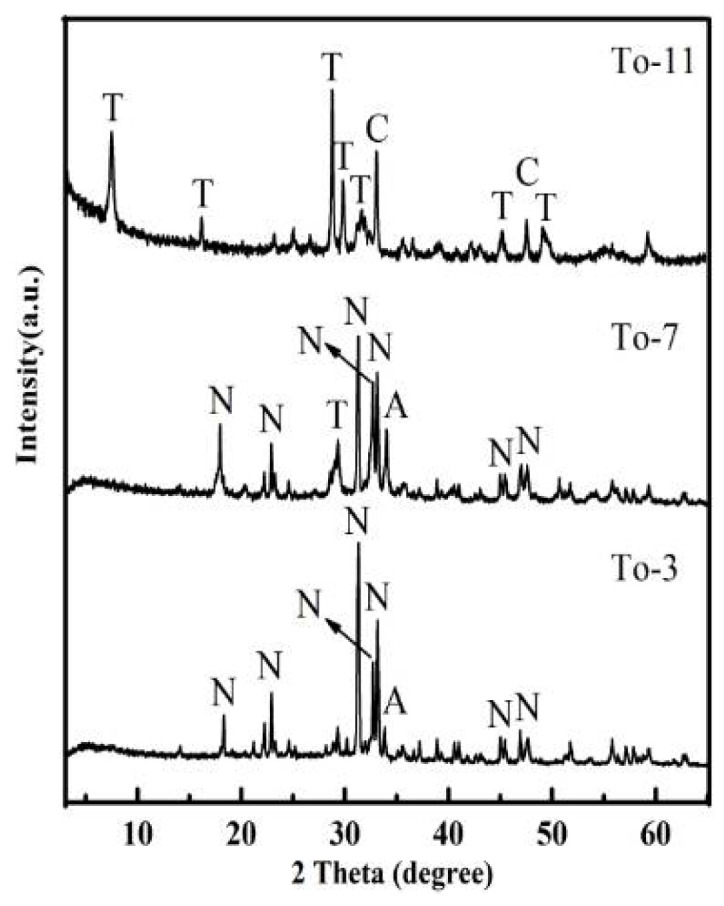
XRD pattern of products synthesized at different temperatures for 12 h. N—NaCaHSiO_4_; A—Ca(OH)_2_; T—11Å-TA; C—Ca_3_Al_2_O_6_.

Powder XRD studies were conducted on the materials obtained with increasing Ca/Si molar ratio at 160 °C (Figure 2). XRD patterns of To-1, To-2, To-3, To-4 and AER were the same except for the peaks height ratio, hence, NaCaHSiO_4_ from AER failed to effectively dissolve and form the calcium silicate hydrate gel (C-S-H) and crystallize to 11Å-TA. The effect of varying reaction temperature from 160 °C to 200 °C was investigated with Ca/Si molar ratio of 1 and treatment time of 12 h (Figure 3). XRD patterns of the samples after hydrothermal reaction in different temperature show a clear decrease in peak intensity corresponding to NaCaHSiO_4_ and Ca(OH)_2_ phases, which almost disappear at higher temperature (200 °C), while 11Å-TA had greater intensity in the main reflections of 7.61°, 5.46° and 28.91°(2*θ*) and Ca_3_Al_2_O_6_ was more clear in the main reflections of 33.13° and 47.72° (2*θ*). Furthermore, attempted synthesize of 11Å-TA at lower hydrothermal temperature was also carried out for comparison. XRD pattern of To-3 was comparable to that of To-7, and the peak intensities of NaCaHSiO_4_ remained almost unchanged, indicating that NaCaHSiO_4_ was stable under hydrothermal temperature of 180 °C. It was also observed that hydrothermal temperature played a major role in the dissolution of Si^4+^ species present in NaCaHSiO_4_ matrix of AER.

**Figure 4:**
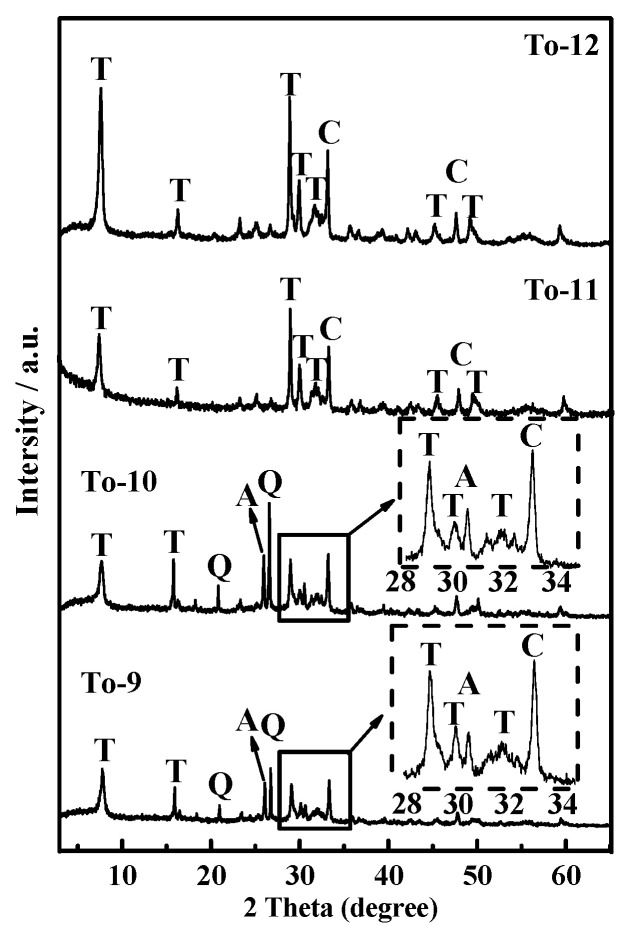
XRD patterns of products synthesized at different Ca/Si molar ratio at 200 °C for 12 h. T—11Å-TA; C—Ca_3_Al_2_O_6_; A—Analcime; Q—Quartz.

XRD patterns of products obtained from the hydrothermal reaction with various Ca/Si molar ratio at 200 °C are shown in Figure 4. The hydrothermal time was fixed at 12 h. The observed peaks of main phases in ARE fully disappeared. Si and Ca were all dissolved and completely used for new mineral formation. All samples show characteristics peaks of 11Å-TA. This highlights the possibility of crystallization of the material over a wider Ca/Si molar ratio range. When the ratio was 0.8, the crystal phases in the product were mainly 11Å-TA, with quartz and analcime detectable only in trace amounts. According to a primitive inference from Hsiao [26], the presence of Na^+^ and a basic solution environment are required for the synthesis of analcime. NaCaHSiO_4_ was sufficiently alkaline to effect the decomposition of itself and produce sufficient SiO_3_^2−^, Na^+^ and alkalinity without addition of any more strongly basic reaction medium during hydrothermal reaction. In addition, some SiO_2_ did not transform into TA but into quartz indicating that Ca source was insufficient. As Ca/Si molar ratio increased, the quartz and analcime characteristic peaks disappeared, while relatively strong reflections of Ca_3_Al_2_O_6_ were detected. According to the above analyses, hydrothermal treatment of AER without addition of extra silica and calcium source formed 11Å-TA. The reactions were expressed as follows:

NaCaHSiO_4_ + OH^−^ → SiO_3_^2−^ +Na + Ca(OH)_2_ (3)

SiO_3_^2−^ + Ca(OH)_2_+H_2_O → C-S-H+OH^−^ (4)

C-S-H → 11Å-Tobermorite (Ca_5_·Si_6_O_16_(OH)_2_·nH_2_O) (5)

**Figure 5:**
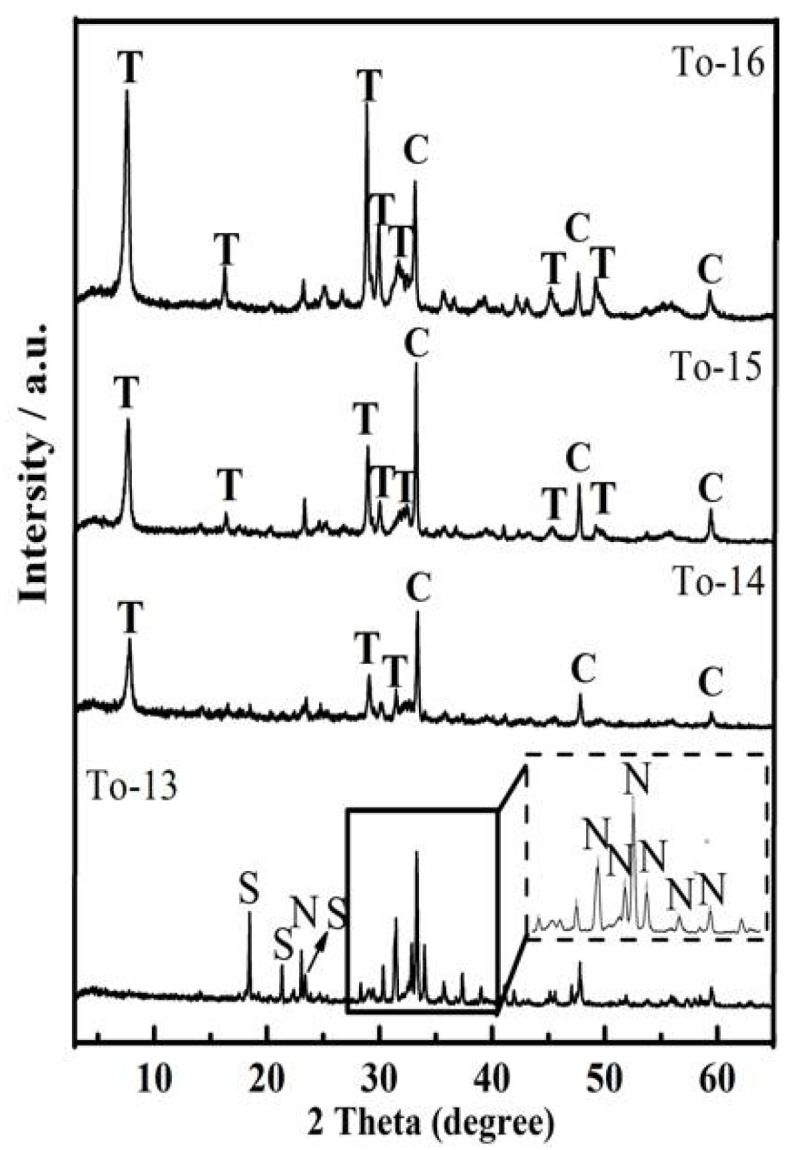
XRD patterns of products synthesized at 200 °C for different time. N—NaCaHSiO_4_; S—Sodium Calcium Aluminum Oxide Silicate Hydrate; T—11Å-TA; C—Ca_3_Al_2_O_6_.

The hydrothermal time plays an important role in the synthesis of 11Å-TA. The effect of varying hydrothermal time between 3 and 12 h was investigated with hydrothermal temperature of 200 °C and Ca/Si molar ratio of 1. Figure 5 presents XRD diffraction pattern of the samples obtained after different hydrothermal treatment times. To-13 signals corresponded to the newly generated sodium calcium aluminum oxide silicate hydrate phase in addition to peaks corresponding to NaCaHSiO_4_ present in the unreacted AER, where most of NaCaHSiO_4_ remained unreactive after 3 h. After which only the characteristic peaks of 11Å-TA were observed at 6 h. However, after 12 h, the main final phase in the product was 11Å-TA; Ca_3_Al_2_O_6_ according to XRD analysis. The patterns remained unchanged, apart from peaks related to 11Å-TA that narrowed as the crystalline phase became more ordered. Hence, the optimal hydrothermal condition was 200 °C for 12 h. The 11Å-TA could be further functionalized to prepare low-cost adsorbing material for water purification.

**Figure 6:**
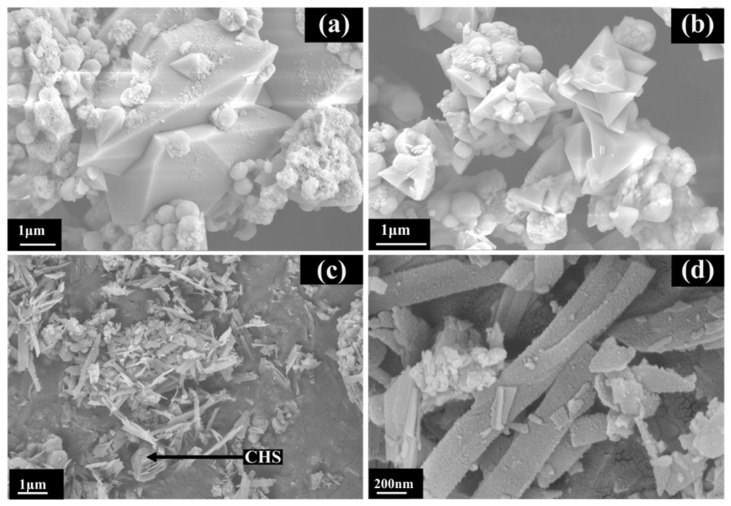
SEM images examining the effect of reaction time on the morphology of the product for (**a**) and (**b**) To-13, (**c**) To-14, (**d**) To-15.

**Figure 7:**
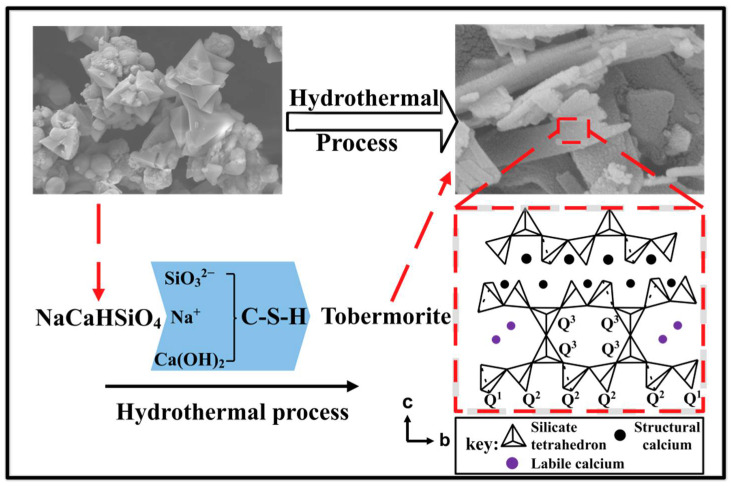
The schematic of the Tobermorite adsorbent (TA) preparation; Q^3^ represents the bridging silicate tetrahedra; Q^2^ represents mid-chain silicate tetrahedra; Q^1^ represents the end group of a chain structure.

Figure 6 shows the morphological changes of the products at different reaction temperatures. As shown in Figure 6a,b, a clear outline of the angular-like crystals microstructure, and particles as irregular globular coexist, which indicate an incomplete reaction and in agreement with XRD results. With the increase in reaction time. Figure 6c shows that as the reaction time increases to 6 h, NaCaHSiO_4_ was no longer present, and To-16 sample displayed a shorter and thinner band-like morphology, with a length of approx 1μm. Additionally, traces of C-H-S phases were observed around 11Å-TA. As shown in Figure 6d, more and more band-like crystallites formed, some of which were coiled together when the reaction proceeded for 9 h. Figure 7 shows the schematic of the TA preparation. The surface area is directly associated with the adsorption capacity for materials using adsorbent. The surface area of TA-adsorbent was determined as 16.77 m^2^·g^−1^ by single-point BET analysis.

### 3.2. Heavy Metal Removal Studies

#### 3.2.1. Effect of Initial pH

pH of the aqueous system has a dramatic effect on adsorption capacity. Excess H^+^ causes the protonation of TA as well as competition with the toxic metal ions to be adsorbed on the active sites. However, pH of the solution also affects the state of toxic metal ions. To prevent the formation of metal hydroxide precipitates in the aqueous system, the effect of the synthesized TA on the uptake capacity of Pb(II) and Cu(II) was studied in pH range of 1.0–6.0 at 25 °C for 6 h. 

**Figure 8:**
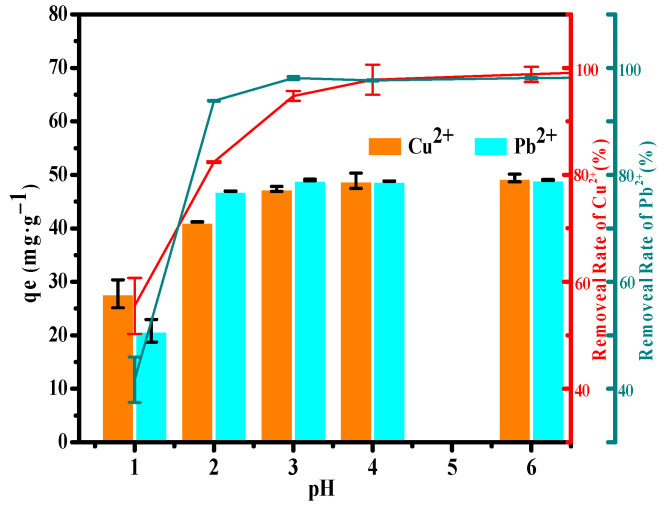
Effect of pH and recycle adsorption performance of TA for Cu(II) and Pb(II). Cation initial concentration was 200 mg·L^−1^, adsorbent dosage was 0.1 g, 25 °C.

The results presented in Figure 8 show that the adsorption capacity of Cu(II) increases with pH from 1 to 3, hence, the adsorption was relatively low under strong acidic conditions. This was because TA surface was positively charged as a result of H^+^ would have the advantage for the competition compare with Cu(II), electrostatic repulsion forces restricted existence of the Cu(II) (dominant species), together with trace amounts of Cu_2_(OH)_2_^2+^ and Cu(OH)^+^ in the proximity of the adsorption interface. Adsorbent surface became deprotonated with increasing pH. Consequently, at pH between 3 and 6, the concentration of OH^−^ increased in order of magnitude, the amount of negative charges on TA surfaces increased via deprotonation of hydroxyl groups, which promoted strong electrostatic attraction forces resulting in removal of Cu(II) ions (>95%). Furthermore, removal rate of Pb(II) adsorbed by TA at different pH values are shown in Figure 8. The same trends were observed for Pb(II) removal efficiencies with increasing pH, which increased the adsorptive efficiency from pH 1.0 to 3.0. At pH < 3, the removal was low owing to the active sites on TA surface being occupied by H^+^. However, the maximum removal reached 98.3% and remained above 97% at pH 3.0–6.0. According to the surface complexation theory, competition between H^+^ and Pb(II) decreased with increasing pH. 

The zero point potential (pH_zpc_) of TA adsorbent is 7.14 at which the net surface charge of the TA adsorbent is equal to zero. When pH below 7.14, it indicating that the adsorbent were negatively charged, which favored Cu(II) and Pb(II) removal from the solution. In addition, the negative surface charge of TA adsorbent accelerates the adsorption rate at the initial stage. Therefore, the obtained results indicated that the alkaline solution medium was favorable for the removal of toxic metal ions.

#### 3.2.2. Effect of Initial Concentration

**Figure 9:**
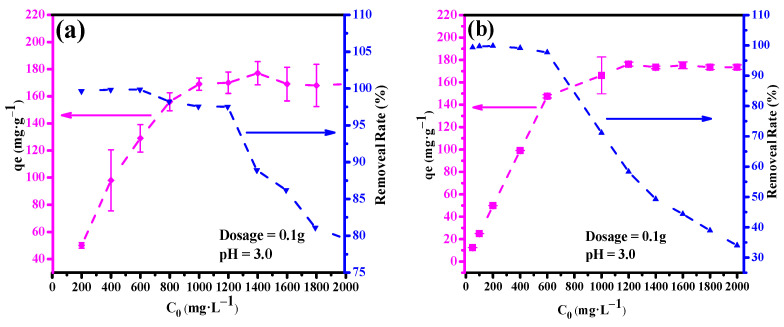
Effect of initial metal ion concentration on adsorption of Cu(II) (**a**) and Pb(II) (**b**) on TA.

As shown in Figure 9, the initial metal ion concentration ranging from 200 mg·L^−1^ to 2000 mg·L^−1^ was examined. Figure 9a depicts that the adsorption capacity of TA increased with Cu(II) concentration. At equilibrium, qe for Cu(II) was achieved by TA at 177.1 mg·g^−1^. This may be attributed to the number of Cu(II) will be less compared to the available sites on TA, resulting in diminished adsorption at low Cu(II) concentration. At higher initial Cu(II) concentration, Cu(II) transfer from the solution to TA was favored. However, as shown in Figure 9a, the removal rate remained over 99% in initial concentration from 200 mg·L^−1^ to 600 mg·L^−1^, but decreased with increasing the initial concentration of Cu(II). As shown in Figure 9b, similar trends were observed for Pb(II), where q_e_ increased with increasing initial concentration and reached a plateau (177.1 mg·g^−1^) at above 1000 mg·L^−1^. The increase of the initial concentration of metal ion enhanced the driving force that overcome mass transfer resistance between the liquid and solid phases. q_e_ reached a plateau due to overlapping of available active sites that adhered the metal ions required for the high concentration of metal ion [27]. Compared with previous reports, the adsorption capacities are significantly higher [28,29], the adsorption capacities are significantly higher. Therefore, TA could effectively treat Cu and Pb-containing wastewater.

#### 3.2.3. Adsorption Isotherm

**Figure 10:**
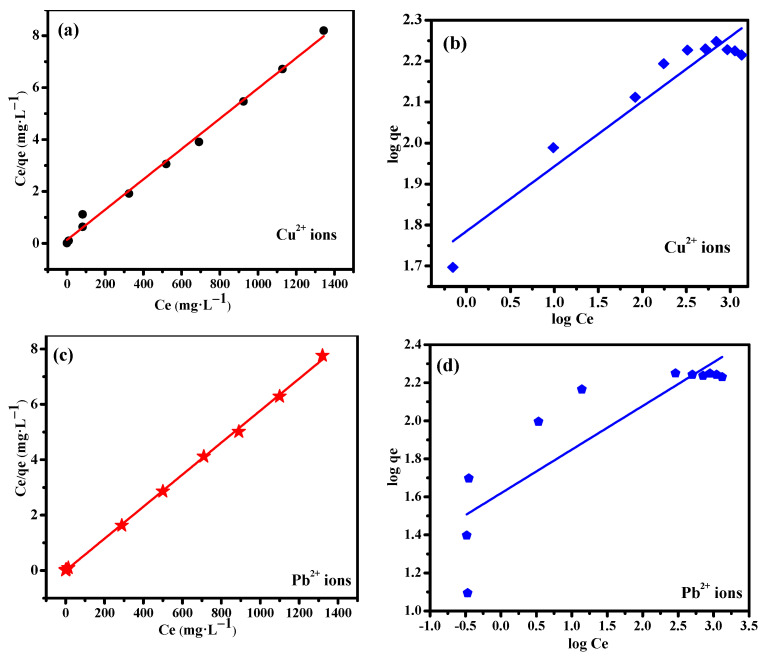
(**a**) Langmuir isotherm; (**b**) Freundlich isotherm (adsorption conditions: Ce (Cu(II)) = 200-2000 mg·L^−1^, V = 10 mL, T = 25 °C, t = 360 min, pH = 5.0, and adsorbent amount = 0.1 g). (**c**) Langmuir isotherm; (**d**) Freundlich isotherm (adsorption conditions: Ce (Pb(II)) = 200-2000mg·L^−1^, V = 10 mL, T = 25 °C, t = 360 min, pH = 5.0, and adsorbent amount = 0.1 g).

The effect of initial concentration on the equilibrium adsorption capacity of metal ion adsorbed on TA was evaluated at 25 °C. The linear forms of the Langmuir (Equation (6)) and Freundlich (Equation (7)) isotherms described the relationship of Cu(II) and Pb(II) in the solid and liquid phase at equilibrium.

(6) $$\begin{array}{c} \mathrm{Langmuir}\;\mathrm{model}:\\ \frac{C_{e}}{q_{e}}=\frac{1}{q_{m}b_{L}}+\frac{C_{e}}{q_{m}} \end{array}$$

(7) $$\begin{array}{c} \mathrm{Freundlich}\;\mathrm{model}:\\ {\mathrm{Logq}}_{e}={\mathrm{LogK}}_{F}+\frac{1}{n}{\mathrm{LogC}}_{e} \end{array}$$

where C_e_ and q_e_ is consistent with it mentioned above; q_m_ is the maximum adsorption capacity of metal ion adsorbed per unit weight of TA; K_F_ and 1/n are the Freundlich constants; b_L_ is the Langmuir constant. The linear plots of the Langmuir and Freundlich isotherms are shown in Figure 10, and the corresponding parameters are calculated and listed in Table 3. Figure 10a,b shows that, the Langmuir model (R_2_ = 0.993) fitted better to the Cu(II) adsorption data compared to the Freundlich model (R_2_ = 0.921). This indicated that the adsorption of Cu(II) ions on TA adsorbent involved a monolayer coverage process. Additionally, the maximum value of q_m_ of TA adsorbent for Cu(II) ions was 171.2 mg·g^−1^ which was comparable to the experiment value (177.1 mg·g^−1^). The separation factor constant (R_L_) was used to evaluate the applicability of the adsorbent to the studied adsorbate [30,31], which was calculated from Langmuir isotherm described in Equation (8).

(8) $$\begin{array}{c} \mathrm{Separation}\;\mathrm{factor}:\\ R_{L}=\frac{1}{1+K_{L}C_{0}} \end{array}$$

where C_o_ denotes the initial concentration. K_L_ values were obtained from the Langmuir isotherm model. As shown in Table 3, R_L_ was between 0.525 and 0.155, and the range from 0 to 1 means that the adsorption process of Cu(II) was theoretically favorable [32].

Figure 10c,d shows the experimental adsorption data of Pb(II) for TA that fitted better using the Langmuir isotherm model (R_2_ = 0.999) compared to the Freundlich model (R_2_ = 0.732). Hence, the active sites of TA were homogeneously distributed on the surface, and Pb(II) exhibited a more preferred monolayer adsorption. The observed better fitted data also indicated weak interaction between active sites of the adsorbents. The maximum adsorption capacity of Pb(II) for TA obtained from the Langmuir isotherm model was 173.01 mg·g^−1^, which was similar to the maximum adsorption capacity. Table 3 displays the separation factor (R_L_), where adsorption was favorable (0 < RL = (0.0135~0.0003) < 1). Therefore, cost-effectiveness and excellent adsorption performance shows that TA is a promising candidate for the removal of Cu(II) and Pb(II) from wastewater.

#### 3.2.4. Effect of Time and Adsorption Kinetics Analysis

**Figure 11:**
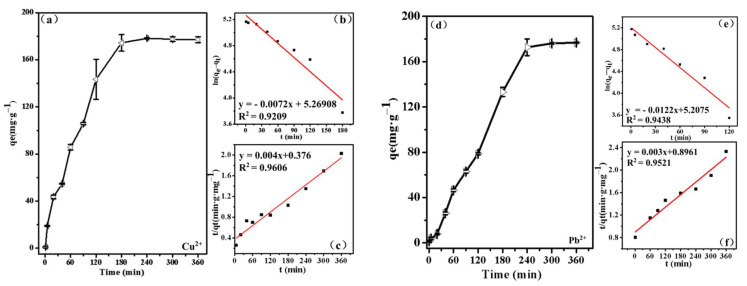
The effect of contact time on the adsorption of Cu(II) ions by TA (**a**); pseudo-fist-order model (**b**) and pseudo-second-order model (**c**); the effect of contact time on the adsorption of Pb(II) ions by TA (**d**); pseudo-fist-order model (**e**) and pseudo-second-order model (**f**).

The rate-controlling steps are essential in order to further elucidate the removal mechanism of Cu(II) and Pb(II) by TA. The kinetic data were fitted to either the pseudo-fist-order (Equation (9)) or pseudo-second-order (Equation (10)) kinetic models.

(9) $$\mathrm{Pseudo}\text{-}\mathrm{first}\text{-}\mathrm{order}\;\mathrm{model}:\;\mathrm{Ln}(q^{e}-q^{t})=Lnq^{e}-K_{1}t$$

(10) $$\mathrm{Pseudo}\text{-}\sec\mathrm{ond}\text{-}\mathrm{model}:\;\frac{t}{q_{t}}=\frac{t}{q_{e}}+\frac{1}{q_{e}^{2}k_{2}}$$

where q_e_ and q_t_ are the adsorption capacities of metal ions at equilibrium and different time, respectively; k_1_ and k_2_ are the rate constants for the pseudo-fist-order model and pseudo-second-order model, respectively. The effect of contact time on the adsorption of Cu(II) by TA is exhibited in Figure 11a. The adsorption process was considerably faster in the first few min and reached equilibrium after 180 min. As shown in Figure 11b,c, the adsorption kinetics of Cu(II) ions on TA was well fitted by both the pseudo-fist-order model (R_2_ = 0.9209) and pseudo-second-order (R_2_ = 0.9606). The simulation results showed that the calculated q_max_ of the pseudo-second-order model was in agreement with the experimental data. Therefore, the adsorption of Cu(II) ions on TA was mainly controlled by chemisorption. In addition, the effects of the contact time on the adsorption of Pb(II) were also investigated (Figure 11d). The parameters of the models were better fitted to the pseudo-second-order (R_2_ = 0.9521) than the pseudo-fist-order model (R_2_ = 0.9438), which indicated the chemisorption was the dominant control step for the adsorption process of Pb(II). k_2_ for Cu(II) was higher than that of Pb(II) and indicated that Cu(II) was adsorbed at a faster adsorption rate, which was consistent with the time required for reaching equilibrium.

Compared with other adsorbents, the biggest advantage of TA as adsorbent is lower costs, as shown in Table 4, several solid waste based low cost adsorbents have been used for the removal of Cu(II) and Pb(II) in previous studies. In general, the removal capacity of TA adsorbent for Cu(II) and Pb(II) compares favorably with those of other solid waste based adsorbents under single metal ions batch conditions.

#### 3.2.5. Adsorption Thermodynamic

Thermodynamic analysis is a scientific theory to study the effect of temperature on the adsorption process. In this study, the temperature range was 25 °C, 45 °C and 65 °C to calculate thermodynamic parameters, actual indicators including Gibbs free energy (ΔG_0_) are deduced from Equations (11) and (12), similarly, the enthalpy (ΔH_0_) and entropy (ΔS_0_) are calculated from the following Equation (13) (Van Hoff’s relation) and are summarized in Table 5.

(11) $$K_{C}=q_{e}{/c}_{e}$$

(12) $${\Delta G}^{0}={-\mathrm{RTlnK}}_{C}$$

(13) $${\mathrm{lnK}}_{C}={\Delta S}^{0}/R-{\Delta H}^{0}/\mathrm{RT}$$

where q_e_ represents the adsorption capacity of the adsorbent at equilibrium state; ce represents the adsorption equilibrium concentration of metal ions in the solution. R is the gas constant, and T is the temperature (K).

Table 5 indicate that the values of ΔG_0_ for Cu(II) and Pb(II) at lower temperature are slightly positive. It expresses that the adsorption process was simple but nonspontaneous at lower temperature. The negative values of ΔH_0_ indicate that Cu(II) and Pb(II) adsorption processes follow the exothermic reaction. Besides, the negative values of ΔS_0_ elucidate a decrease in the degree of randomness of Cu(II) and Pb(II) at the solid adsorbate interface of the TA adsorbent. 

## 4. Conclusions

Using reside after extracting aluminum as raw material, we developed a low-cost TA via hydrothermal process. The experiments were performed under various conditions to determine optimal operation parameters and obtain well crystallized TA within 12 h at 200 °C without addition of extra silica and calcium source. Moreover, synthesized TA was employed to remove toxic substances (Cu(II) and Pb(II)) from wastewater. The adsorption data was in good agreement with the Langmuir model, which indicated that adsorption occurred in a homogeneous adsorption site via a monolayer reaction and maximum adsorption obtained at capacity of 177.1 and 176.2 mg·g^−1^ for Cu(II) and Pb(II), respectively. The kinetic study showed that adsorption followed the pseudo-second-order model, indicating a chemisorption process. Thermodynamic analysis showed the adsorption of Cu(II) and Pb(II) by synthesized TA adsorbent is a nonspontaneous and exothermic process. Therefore, the prepared TA adsorbent has potential application in treatment of heavy metal pollutants. Furthermore, Excellent adsorption properties of adsorbent are not only attribute required, its regeneration is a critical factor as well. For this reason, the regeneration studies of TA adsorbent is a scientific problem worthy of further attention.

## Figures and Tables

**Table 1:** Chemical composition of AER (ωt%).

Component	CaO	SiO_2_	Al_2_O_3_	Na_2_O	Fe_2_O_3_	TiO_2_	MgO	Others
Mass (%)	37.39	35.57	2.10	17.91	2.03	2.76	1.86	0.39

**Table 2:** Ranges and values of the experimental parameters for 11Å-TA synthesis using AER.

Samples Label	Experiment Conditions			Liquid-Solid Ratio (mL·g^−1^)
	Ca/Si (Ratio)	Temperature (°C)	Time (h)	
To-1	0.8	160	12	25
To-2	0.9			
To-3	1.0			
To-4	1.1			
To-5	0.8	180		
To-6	0.9			
To-7	1.0			
To-8	1.1			
To-9	0.8	200		
To-10	0.9			
To-11	1.0			
To-12	1.1			
To-13	1.0 ^a^	200	3	
To-14			6	
To-15			9	
To-16			12	

^a^ refers to suitable parameter values selected from previous set to be applied for the next sets of experiments.

**Table 3:** Comparison between Langmuir and Freundlich constants by linear fitting for TA at 25 °C.

Kinetic Model	Parameter	Metal Ions	
		Cu(II)	Pb(II)
Langmuir	q_m_ (mg·g^−1^)	171.2	173.01
	b_L_(min^−1^)	0.00452	1.4522
	R_2_	0.993	0.999
	R_L_	0.525~0.155	0.0135~0.0003
Freundlich	1/n	0.158	0.229
	K_F_ (L·mg^−1^)	60.94	41.591
	R_2_	0.921	0.732

q_m_ is the maximum adsorption capacity of metal ion; b_L_ is the langmuir constant; R_L_ is the separation factor constant; K_F_ and 1/n are the freundlich constants.

**Table 4:** Comparison of adsorption capacity of Cu(II) and Pb(II) by TA and other adsorbents.

Adsorbents	q_m_ (mg·g^−1^)		Reference
	Cu(II) Sorbed	Pb(II) Sorbed	
Magnetic attapulgite composites	189.0	142.8	[33]
Novel geogolymers based on coal gangue and red mud	90.0	137.7	[29]
Tetrazole-bonded bagasse	253.5	89.3	[34]
Zeolites prepared from CFA	57.8	109.9	[35]
Linde F(K) zeolite from CFA	18.5	46.5	[36]
Activated bentonite	9.7	21.3	[37]
TA-adsorbent	177.1	176.2	This study

**Table 5:** Thermodynamic parameter of Cu(II) and Pb(II) adsorption by the obtained TA adsorbents.

Metal	T (K)	K_c_	ΔG_0_ (kJ·mol^−1^)	ΔH_0_ (KJ·mol^−1^)	ΔS_0_ (J·mol^−1^·K^−1^)	R (kJ·mol^−1^·K^−1^)
Cu(II)	298.15	0.171	4.368	−1.090	−9.047	8.3 × 10^−3^
	318.15	0.156	4.906			
	338.15	0.144	5.439			
Pb(II)	298.15	0.173	4.342	−2.760	−5.277	
	318.15	0.155	4.923			
	338.15	0.132	5.683			

## Data Availability

Not applicable.
